# Burnout and Back Pain and Their Associations With Homecare Workers' Psychosocial Work Environment—A National Multicenter Cross‐Sectional Study

**DOI:** 10.1111/jan.16931

**Published:** 2025-04-02

**Authors:** Tania Martins, Michael Simon, Nathalie Möckli, Carla Meyer‐Massetti, Roland Fischer, Christine Serdaly, Franziska Zúñiga

**Affiliations:** ^1^ Institute of Nursing Science, Department of Public Health University of Basel Basel Switzerland; ^2^ Clinical Pharmacology and Toxicology, Department of General Internal Medicine Inselspital—University Hospital of Bern Bern Switzerland; ^3^ Institute of Primary Health Care (BIHAM) University of Bern Bern Switzerland; ^4^ Centre for Primary Health Care University of Basel Liestal Switzerland; ^5^ Serdaly & Ankers Snc Conches Switzerland

**Keywords:** burnout, home care agencies, home health nursing, low back pain, occupational health

## Abstract

**Aims:**

To determine the prevalence of burnout and back pain in homecare workers in Switzerland and assess their associations with psychosocial work environment factors.

**Design:**

National multicentre cross‐sectional study.

**Methods:**

Using paper‐pencil questionnaires, data were collected from January 2021 to September 2021 from employees of 88 homecare agencies across Switzerland. Respondents who identified themselves as administrators, apprentices, or trainees, who were in leadership positions, or who were not involved in the provision of care or housekeeping were excluded from this analysis. Burnout was assessed with the Copenhagen Burnout Inventory Scale (possible score range 0–100) and back pain with a single item from the Federal Statistical Office's Swiss Health Survey. Multilevel regression analyses were used to assess burnout and back pain's associations with psychosocial work environment factors.

**Results:**

We included 2514 homecare workers. More than two‐thirds (68.6%) reported back pain in the past 4 weeks. The overall mean burnout score was 36.0 (SD 18.3). Poorer work‐life balance, higher perceived workload and verbal aggression from clients were positively associated with both outcomes. Better leadership and social support from colleagues were negatively associated with burnout. Higher role conflict levels correlated with higher burnout levels.

**Conclusion:**

Our findings indicate that the psychosocial work environment should be considered when designing interventions to reduce the prevalence of burnout and back pain among homecare workers.

**Implications for the Profession and Patient Care:**

The high reported burnout and back pain prevalences among homecare workers highlight an urgent need to design and implement psychosocial work environment‐improving interventions. In addition to contributing to homecare employees' long‐term attraction and retention, protecting and promoting their health and well‐being will likely not only benefit them, but also contribute to patient safety, quality of care and homecare sustainability.

**Impact:**

The study reports the prevalence of burnout and back pain among homecare workers and their associations with psychosocial work environment factors. The results indicate that six psychosocial work environment factors—work‐life balance, perceived workload, leadership quality, levels of social support from colleagues, role conflict levels, and verbal aggression from clients—all correlate with burnout and/or back pain in homecare workers. For policy makers, researchers, healthcare managers, and homecare agencies, this study's findings will inform the development of interventions to enhance homecare work environments, leading to improvements both in workers' health and in the quality of their care.

**Reporting Method:**

We have adhered to the Strengthening the Reporting of Observational Studies in Epidemiology (STROBE) reporting checklist for cross‐sectional studies.

**Patient or Public Contribution:**

Our stakeholder group included patient representatives, policy makers, researchers, clinicians and representatives of professional associations. Throughout the study, all provided support and input on topics including questionnaire development, result interpretation and the design of strategies to improve response rates.

**Summary**

- What does this paper contribute to the wider global clinical community?
  ○ This paper highlights the impact of psychosocial work environment factors on two homecare worker health outcomes: burnout and back pain.
  ○ By identifying factors associated with those outcomes, this research will inform the development of targeted strategies at the individual, healthcare organisation and policy levels.
  ○ Ultimately, by promoting a healthier, more supportive and more attractive work environment, these findings will buttress home healthcare's long‐term sustainability.

## 1 Introduction

Compared to other fields of employment, healthcare workplaces are extremely hazardous. In 2020, U.S. statistics indicated a rate of healthcare work‐related illness and injury twice that of private industry (U.S. Bureau of Labor Statistics 2021). In 2022, a World Health Organization (WHO) report estimated that worldwide, roughly 60 million healthcare professionals were regularly exposed to a wide range of occupational health and safety hazards (World Health Organization 2022). That same year, a WHO report noted that unsafe working conditions—factors that increase the risks of occupational illness, injury and absenteeism among workers—represent an ongoing financial burden of 2% of healthcare expenditures (World Health Organization 2022).

Both the demand for healthcare and the complexity of homecare services are growing. Still, little is known about the psychosocial work environment and occupational health of the workers in this growing sector. To design interventions that promote healthy workplaces in this sector, we need first to assess homecare workers current occupational health, such as the prevalence of back pain and burnout, and second, to identify the psychosocial work environment risk factors that will potentially affect those workers' mental and physical well‐being.

### 1.1 Background

Harm to healthcare professionals can be both physical and psychological. One example of physical harm is back pain. Although not life‐threatening, back pain has a negative impact on healthcare professionals' well‐being. Globally, across all age groups, as a leading cause of disability‐adjusted life‐years (DALYs), back pain ranks fourth most common among all diseases in women and ninth in men (Patwardhan et al. 2024). Back pain in nurses has been linked to physical tasks such as positioning/mobilising patients without mechanical assistance (Santos et al. 2016). In homecare, the availability of technical aids for patient handling, for example, lifters, is limited (Hegewald et al. 2018). In addition, in a study of 181 homecare workers in Norway, Robstad and Westgaard (2014) suggested that tension and musculoskeletal symptoms can also arise from mental stress. Back pain is thus a key indicator for health in the homecare workforce.

As for psychological harm, one example is burnout as defined by the International Classification of Diseases (ICD‐11), an occupational phenomenon resulting from chronic, inadequately managed workplace stress (World Health Organization, 2019/ 2021). Its effects are thought to include diminished performance, including an increased occurrence of errors (Hall et al. 2016). Among nurses, a recent meta‐analysis reported an 11.2% global prevalence of burnout symptoms (Woo et al. 2020). Sustained high levels of work demands, for example, high workload, can decrease motivation and lead to withdrawal from work as a self‐protective mechanism (Demerouti et al. 2001). For example, if high workload prevents otherwise motivated care workers from achieving their goals (e.g., providing good quality care), outcomes might include frustration and decreased motivation. With the increasing demand for homecare services and the growing complexity of care needs, homecare workers may experience high workloads. It is therefore important to assess burnout in this population to better understand its prevalence and identify risk factors.

According to the WHO healthy workplace model, in addition to other factors (physical work environment, enterprise community involvement and personal health resources), a healthy workplace depends on its psychosocial work environment (Burton 2010), that is, psychosocial factors can represent significant occupational hazards for workers.

Healthcare professionals are exposed to various psychosocial work environment risk factors. These include long working hours, shift work, poor leadership, time pressure and understaffing, all of which have been associated with burnout (Dall'Ora et al. 2020). Meanwhile, high workload, low job control and lack of social support have been named as possible predictors of back pain (Buruck et al. 2019).

Homecare‐specific risk factors include the need to work in unpredictable, highly variable, non‐medical settings and environments, often alone in clients' homes. This can entail challenges in obtaining timely access to medical equipment, lack of assistive devices (e.g., for lifting clients) and travel‐related risks en route to clients' homes (Carpenter et al. 2017). Providing care at clients' homes involves additional challenges, such as household distractions, non‐medical requests from clients and a social environment that lacks adequate team support for more cognitively or physically demanding tasks (Hignett et al. 2016). In 2020, the U.S. rate of occupational illnesses and injury among home healthcare workers was 3.3 per 100 full‐time equivalent workers (U.S. Bureau of Labor Statistics 2021). We could find no similar information for home healthcare workers in Europe.

In this context, healthy psychosocial work environments within homecare agencies may be particularly important to counterbalance the unpredictable physical work environments of clients' homes.

## 2 The Study

### 2.1 Aim(s)

This study had two aims: (1) to determine the prevalence of burnout and back pain in homecare workers in Switzerland; and (2) to assess the association between that prevalence and elements of those workers' psychosocial work environments.

## 3 Methods

### 3.1 Design, Setting and Sample

This study is part of the SPOT^nat^ (quality and coordination in homecare) study, a national multicenter cross‐sectional study conducted in 2021 to assess the quality of care and coordination in the Swiss homecare setting. The conceptual framework of the SPOT^nat^ study includes possible influencing factors at the macro, meso, and micro levels. On the meso level, structure quality includes factors such as the workforce, the work environment, and the characteristics of homecare agencies' employees. In this study we focus on the psychosocial work environment and its association with burnout and back pain, as aspects that might affect the quality of care. Using a stratified random sample, SPOT^nat^ recruited 88 homecare agencies across Switzerland surveying employees (*n* = 3223) and managers (*n* = 88), as well as clients (*n* = 1509) and their relatives (*n* = 1168). Further details on the SPOT^nat^ setting, sample and sample size calculations can be found in the SPOT^nat^ study protocol (Möckli et al. 2021). The current study used only homecare agencies' characteristics and employee‐reported data.

In Switzerland, in accordance with varying cantonal and municipal policies, homecare is provided by self‐employed nurses and by homecare agencies, which can be either non‐profit or for‐profit. Homecare includes both nursing and housekeeping. At the time of writing, this field is experiencing the highest annual growth of any branch of the Swiss healthcare sector. In 2022, homecare services were provided across Switzerland by approximately 2700 providers, employing roughly 61,100 workers (an increase of approximately 172% in full‐time equivalents compared to 2002) (Federal Statistical Office 2022).

Within each agency, we included all homecare workers who were at least 18 years old, had been employed by that agency for at least 3 months, were involved directly or indirectly in patient care, and were able to understand written German, French or Italian. We excluded homecare agencies with fewer than 10 employees and self‐employed nurses. For this sub‐analysis, we also excluded employees who identified themselves as administrators, apprentices, or trainees, who were in leadership positions, or who were not involved either in the provision of nursing care or in housekeeping. As we included formal careers with different educational backgrounds (registered nurses (university/college degree, BScN or at least a 3‐year diploma program), licensed practical nurses, certified nursing assistants, and personal care workers), we decided to use the term homecare workers. Respondents with missing information regarding any of the inclusion criteria were excluded.

### 3.2 Variables and Measurements

#### 3.2.1 Health‐Related Outcomes

To assess burnout, we used a personal burnout subscale (the validated Copenhagen Burnout Inventory [CBI])—a six‐item subscale on which individuals report their levels of physical and psychological fatigue and exhaustion. Each item uses a 5‐point Likert‐type scale. For our analysis we converted the scale numbers from 1, 2, 3, 4 or 5 respectively to 0 (‘never/almost never’), 25 (‘seldom’), 50 (‘sometimes’), 75 (‘often’) and 100 (‘always’). For participants who answer at least three items, the scale's authors recommend using the mean of the answered items as the scale score. Higher scores indicate higher levels of burnout. In our sample, the scale showed high internal consistency, yielding a Cronbach's alpha of 0.90 (95% CI; 0.89–0.90). Recognising that many authors categorise burnout levels simply as either ‘low’ (< 50) or ‘high’ (≥ 50), we reported both the mean scores and prevalence of responses in these two categories, with ‘low’ corresponding to mean scores of 0 to 49 and ‘high’ corresponding to scores of 50 to 100.

Back pain was assessed with a single item from the Federal Statistical Office's Swiss Health Survey. That item asked whether the workers had experienced back pain in the past 4 weeks. The answer options were 1 (‘not at all’), 2 (‘a little’) and 3 (‘strong’). For our analysis, we dichotomized the answer options as 0 (‘no’ for ‘not at all’) and 1 (‘yes’ for ‘a little’ and ‘strong’).

#### 3.2.2 Psychosocial Work Environment

Leadership was measured using the Practice Environment Scale of the Nursing Work Index's (PES‐NWI's) Nurse Manager Ability, Leadership and Support of Nurses subscale. This subscale contains five items assessing the extent to which the supervisor is supportive, uses mistakes as learning opportunities (rather than the basis of harsh criticism or punishment), praises and recognises work done well, supports workers' decision‐making, and is an overall good manager and leader. With all items' response options ranging from 1 (‘strongly disagree’) to 4 (‘strongly agree’), that is, higher scores representing more favourable leadership, the scale is scored using the mean of the five items' responses. In our sample, the Cronbach's alpha for this subscale's scores was 0.87 (95% CI; 0.86–0.88).

Both role conflicts and social support from colleagues were measured using subscales of the Copenhagen Psychosocial Questionnaire (COPSOQ). On a 5‐point Likert scale, the COPSOQ's possible responses range from 0 (‘never/hardly ever’) to 4 (‘always’). With two items, the role conflicts subscale assesses whether conflicting demands are present at work and whether the workers feel they are required to perform tasks differently than they should. Our sample's Cronbach's alpha was 0.70 (95% CI; 0.67–0.73). Scoring this subscale requires calculating the mean of the two items, with higher scores indicating a higher incidence of role conflicts, that is, lower scores are more desirable. The social support from colleagues subscale includes two items, assessing how often workers receive needed help and support. The Cronbach's alpha for our sample was 0.62 (95% CI; 0.58–0.65). This subscale is scored using the mean of the two items' ratings, with higher scores indicating more perceived social support from colleagues.

Perceived workload was measured using the National Aeronautics and Space Administration Task Load Index (NASA‐TLX). Using ratings on a 20‐point scale ranging from 1 (‘low’) to 20 (‘high’) (or, for performance, ‘poor’ to ‘good’), this scale measures six dimensions (mental demands, physical demands, temporal demands, performance, effort and frustration) regarding the last shift worked. Total perceived workload was calculated as the unweighted mean score of the six NASA‐TLX items (scaled to 0–100), with higher values indicating higher perceived workload. Our sample's Cronbach's alpha was 0.64 (95% CI; 0.62–0.66).

Work‐life balance was assessed using the work‐life climate scale. This contains seven items on how often, over the last 7 days, employees skipped a meal, ate a poorly balanced meal, worked without a break, arrived home later than planned, changed personal plans because of work, had difficulty sleeping and slept less than 5 h in a night. The 4‐point response scale for each work‐life climate item begins with 1 (‘rarely or none of the time (< 1 day)’), followed by 2 (‘some or a little of the time (1–2 days)’), 3 (‘occasionally or a moderate amount of time (3–4 days)’) and 4 (‘all of the time (5–7 days)’). The score is calculated as the mean of the seven items, with higher scores reflecting poorer work‐life balance. The Cronbach's alpha in our sample was 0.75 (95% CI; 0.73–0.77).

Overtime was measured with a single 5‐point item adapted from the Registered Nurse Forecasting (RN4CAST) study to assess how frequently employees work more than 30 min of overtime. Answer options are 1 (‘almost every shift’), 2 (‘every 2–4 working days’), 3 (‘every 5–7 working days’), 4 (‘less frequently’) and 5 (‘never’). For analysis, we dichotomized this variable into 0 (‘never’ and ‘less frequently’) and 1 (‘almost every shift’, ‘every 2–4 working days’, 3 ‘every 5–7 working days’), that is, lower scores indicate more frequent overtime.

For verbal violence, we used a self‐developed item to assess how often homecare workers experienced verbal violence from clients over the past year. Response options ranged from 0 (‘never’) to 6 (‘every day’) on a 7‐point Likert scale. For this analysis, the answer options were dichotomized into 0 (‘never’ [0]) and 1 (‘at least once’ [options 1–6]).

In addition, we assessed whether the homecare agency offered flexible working schedules. This was gauged using a single item on the homecare agency questionnaire completed by the managers. The response options were (‘no’) and (‘yes’).

#### 3.2.3 Workers' Characteristics

For workers' socio‐demographic characteristics, we collected data on age (in years), gender (‘male’, ‘female’, ‘non‐binary’), percentage of employment, job category (dichotomized into (‘registered nurses’) and (‘all other nursing and care staff with lower levels of training’)), and years of professional experience in the homecare agency where they were currently working (in years).

Overall job satisfaction was assessed as a single variable with a single item from the COPSOQ, with response options ranging from 1 (‘very dissatisfied’) to 4 (‘very satisfied’) on a 4‐point Likert scale.

#### 3.2.4 Homecare Agencies' Characteristics

To characterise homecare agencies, we collected data on their profit status (non‐profit, for‐profit), language region (German, French, Italian) and size (number of full‐time equivalent positions).

### 3.3 Data Collection

Data collection took place from January 2021 to September 2021, with each agency choosing when to begin the 9‐week data collection process. Paper‐pencil questionnaires (in German, French or Italian) were distributed by a local facilitator to all homecare workers who met the inclusion criteria.

### 3.4 Data Analysis

We calculated descriptive statistics. These included frequencies, percentages, means (and standard deviations) or medians (and interquartile ranges).

For aim 1, to assess the prevalence in our sample of our target outcomes—burnout and back pain—we conducted descriptive analyses. For burnout, we reported the mean score on the CBI scale; for back pain, we used the percentage of workers who reported experiencing it over the last 4 weeks.

For aim 2, to explore psychosocial work environment factors' relationships with burnout and back pain, we ran two separate regressions (one for each outcome). In preparation, we assessed the cluster effect by calculating the intraclass correlation coefficient (ICC) (1) for each. For burnout, the ICC (1) was 0.08 (95% CI; 0.05–0.11); for back pain, it was 0.03 (95% CI; 0.01–0.04). Because the sample included groups of homecare workers nested within diverse homecare agencies (e.g., regarding size, language region, ownership) we ran multilevel models.

To calculate the ICC (1), we used the R statistical software ‘rptR’ package. First, we ran bivariate (two‐variable) regression models pairing each psychosocial environment factor with each outcome to assess their associations. Next, using the ‘lme4’ package, we ran multivariate regression models that included all psychosocial work environment factors. For the burnout outcome, we ran multilevel linear regressions. For back pain, we ran multilevel logistic regressions. As a third step, we added four individual factors—age, gender, job category and job satisfaction—as control variables.

To ensure that there was low or no multicollinearity between the independent variables, we checked the variance inflation factors (VIFs), considering VIFs < 5 acceptable (Thompson et al. 2017). In fact, the VIFs were all < 2. Considering that figures below 1.5 are generally treated as non‐multicollinear, this result was very low.

To determine the model fit, we used the Akaike information criterion (AIC) (Cavanaugh and Neath 2019) and Nakagawa's *R* ^2^ (Nakagawa and Schielzeth 2013), particularly the ‘performance’ package. A *p*‐value of < 0.05 was considered significant. Coefficient estimates (*B*) or odds ratios (OR), and 95% confidence intervals (CI) were reported as appropriate. And to check whether treating the back pain outcome as ordinal would change the model's conclusions, we conducted a sensitivity analysis. To do so, using the R ‘ordinal’ package, we ran ordinal logistic regressions.

The proportion of missing data ranged from 0% to 3.3%. One variable (work experience) had a higher level of missing data (7.0%); however, this was only used to describe the sample. Consequently, listwise deletion was deemed an appropriate approach.

All analyses were conducted in R version 4.2.3 for MacOS. The references for all instruments and R packages used are provided in Appendix S1.

### 3.5 Ethical Considerations

The Ethics Committee of Northwestern and Central Switzerland (EKNZ) issued a Declaration of No Objection (Req‐2020‐00110). Each paper‐pencil questionnaire included information explaining the study's purpose, describing the measures taken to protect participants' anonymity and emphasising the voluntary nature of participation. To ensure anonymity, a stamped, pre‐addressed envelope was included to return the questionnaire directly to the study team. To further ensure confidentiality, each agency's questionnaire set identified the agency only via a unique code. The key to these codes was only accessible to the study team's core members. Completion and submission of the questionnaire were considered to indicate informed consent.

## 4 Results

The questionnaire was completed by 3223 homecare workers. After the application of inclusion/exclusion criteria, data from 2514 homecare workers remained for analyses (see Appendix S2).

### 4.1 Sample Characteristics

Our sample originated from 88 homecare agencies, of which 70.5% (*n* = 62) were non‐profit. Of the 88 agencies, 67 (76.1%) were located predominantly in Switzerland's German–speaking region, followed by the French‐ (15.9%, *n* = 14) and Italian‐speaking regions (8.0%, *n* = 7). Agencies had a median of 25.0 (IQR 12.9–53.1) full‐time equivalents.

Most participating homecare workers were female (94.4%), were aged a mean of 46.8 years (SD 11.8) and worked a mean percentage of employment of 62.4% (SD 22.0) (see Table 1). More than one‐third (38.5%) were registered nurses. On average, respondents had worked in their agencies for 6.8 years (SD 6.9).

**TABLE 1:** Descriptive statistics of sample characteristics, independent and dependent variables (*n* = 2514).

Variables	*n* (%)	Mean (SD)	Missing *n* (%)
Individual factors			
Age (years)		46.8 (11.8)	72 (2.9)
Gender			
Female	2365 (94.4)		10 (0.4)
Male	134 (5.4)		
Non‐binary	5 (0.2)		
Percentage of employment (%)		62.4 (22.0)	63 (2.5)
Job category			
Registered nurses	969 (38.5)		0 (0)
Other nursing and care staff^a^	1545 (61.5)		
Work experience in this homecare agency (years)		6.8 (6.9)	175 (7.0)
Overall job satisfaction (scale 1–4)		3.1 (0.5)	82 (3.3)
Health‐related outcomes			
Burnout (scale 0–100)		36.0 (18.3)	12 (0.5)
Back pain in the past 4 weeks			
Yes	1701 (68.6)		36 (1.4)
No	777 (31.4)		
Psychosocial work environment factors			
Leadership (scale 1–4)		3.3 (0.6)	44 (1.8)
Social support from colleagues (scale 0–100)		61.7 (21.3)	29 (1.2)
Role conflicts (scale 0–100)		30.3 (21.6)	30 (1.2)
Work‐life balance (scale 1–4)		1.7 (0.5)	18 (0.7)
Perceived workload (scale 1–20)		10.3 (2.8)	18 (0.7)
Overtime			
Yes (weekly)	1441 (58.2)		39 (1.6)
No (never/less frequently)	1034 (41.8)		
Verbal aggression from clients (past year)			
Yes	1742 (69.7)		13 (0.5)
No	759 (30.3)		
Homecare agency offers flexible working schedules			
Yes	1582 (62.9)		0 (0)
No	932 (37.1)		

Abbreviations: *n*, number; SD, standard deviation.

^a^ Of the total sample, 28.9% were licensed practical nurses, 23.9% were certified nursing assistants, and 8.7% were personal care workers.

### 4.2 Prevalence of Burnout and Back Pain

The mean personal burnout score was 36.0 (SD 18.3) (see Table 1). Slightly more than a quarter (26.3%, *n* = 657) of all respondents reported a burnout score ≥ 50. Most homecare workers reported back pain (68.6%) in the past 4 weeks.

### 4.3 Psychosocial Work Environment Factors

Participating homecare workers rated their leadership with a mean score of 3.3 (SD 0.6) on a scale of 1–4, social support from colleagues with a mean score of 61.7 (SD 21.3) out of 100, and role conflicts with a mean score of 30.3 (SD 21.6) out of 100 (see Table 1). More than half (58.2%) reported working more than 30 min overtime in the last week. Most (62.9%) worked in agencies that offered flexible working schedules. Rated on a scale of 1–4, the mean work‐life balance score was 1.7 (SD 0.5).

### 4.4 Association Between Burnout and Psychosocial Work Environment Factors

The unadjusted multivariable linear regression model linked all factors except overtime with burnout (see Table 2). Adjusted for individual factors (control variables), the final model showed several significant associations. Better leadership (*B* = −2.63, 95% CI [−3.83, −1.43]) and better social support from colleagues (*B* = −0.03, 95% CI [−0.06, −0.00]) were negatively associated with burnout. Higher levels of role conflict (*B* = 0.04, 95% CI [0.01, 0.07]), poorer work‐life balance (*B* = 10.73, 95% CI [9.40, 12.06]), higher perceived workload (*B* = 1.34, 95% CI [1.09, 1.59]), and verbal aggression from clients in the past year (B = 1.73, 95% CI [0.37, 3.08]) were positively associated with burnout.

**TABLE 2:** Results of the linear regression models of the psychosocial work environment factors and their associations with burnout (*n* = 2224).

Variables	Burnout bivariate models	Burnout multivariable model with psychosocial work environment variables (unadjusted model)	Burnout multivariable model with psychosocial work environment variables and individual factors (control variables) (adjusted model)
	*B* [95% CI]	*B* [95% CI]	*B* [95% CI]
Intercept		15.12*** [9.51, 20.73]	38.48*** [31.57, 45.38]
Psychosocial work environment			
Leadership	−8.93*** [−10.18, −7.70]	−4.03*** [−5.21, −2.85]	−2.63*** [−3.83, −1.43]
Social support from colleagues (scale 0–100)	−0.11*** [−0.15, −0.08]	−0.05** [−0.08, −0.02]	−0.03* [−0.06, −0.00]
Role conflicts (scale 0–100)	0.21*** [0.17, 0.24]	0.06*** [0.02, 0.09]	0.04** [0.01, 0.07]
Work‐life balance (scale 1–4)	16.67*** [15.41, 17.92]	11.84***[10.47, 13.18]	10.73*** [9.40, 12.06]
Perceived workload (scale 1–20)	2.69*** [2.44, 2.94]	1.45*** [1.20, 1.70]	1.34*** [1.09, 1.59]
Overtime: yes	7.00*** [5.47, 8.54]	−0.42 [−1.79, 0.96]	0.16 [−1.21, 1.53]
Verbal aggression from clients: yes	6.97*** [5.37, 8.58]	1.97** [0.59, 3.37]	1.73* [0.37, 3.08]
Offers flexible working schedules	−3.63* [−6.61, −0.66]	−2.46* [−4.70, −0.25]	−2.02 [−4.16, 0.11]
Individual factors			
Age (years)	−0.21*** [−0.27, −0.14]		−0.14*** [−0.20, −0.09]
Gender: male^a^	−2.94 [−6.19, 0.30]		−4.43*** [−7.05, −1.81]
Gender: non‐binary^a^	5.80 [−14.34, 25.92]		−9.62 [−25.79, 6.54]
Job category: RN^b^	1.39 [−0.15, 2.93]		−1.90** [−3.19, −0.62]
Overall job satisfaction (scale 1–4)	−12.76*** [−14.06, −11.47]		−5.89*** [−7.16, −4.62]
Random effect			
Homecare agencies (variance [SD])		10.91 [3.30]	9.79 [3.13]
Effect size			
AIC		18,292	18,163
Marginal *R* ^2^		0.336	0.376
Conditional *R* ^2^		0.369	0.405

*Note:* α‐level for significance denoted by asterisk(s).

Abbreviations: AIC, Akaike information criterion; *B*, unstandardised regression coefficients; CI, confidence interval; RN, registered nurses; SD, standard deviation.

^a^ Female.

^b^ All other nursing and care staff with lower levels of training.

* *p* < 0.05.

** *p* < 0.01.

*** *p* < 0.001.

Of the individual factors (control variables), age (older) (B = −0.14, 95% CI [−0.20, −0.09]), gender (male) (B = −4.43, 95% CI [−7.05, −1.81]), job category (RN) (B = −1.90, 95% CI [−3.19, −0.62]) and (higher) job satisfaction (B = −5.89, 95% CI [−7.16, −4.62]) were negatively associated with burnout. The final model explained 40% of the variance in personal burnout.

We provide a subgroup analysis for the different job categories (‘registered nurses’ and ‘other nursing and care staff’) in Appendix S3.

### 4.5 Association Between Back Pain and Psychosocial Work Environment Factors

In the unadjusted multilevel logistic regression model, poorer work‐life balance, higher perceived workload and verbal aggression from clients were positively associated with back pain (see Table 3). When the individual factors were added to the model (adjusted model), the same predictors remained significant, that is, work‐life balance (OR 1.62, 95% CI [1.29, 2.02]); perceived workload (OR 1.07, 95% CI [1.03, 1.12]); and verbal aggression from clients (OR 1.34, 95% CI [1.09, 1.65]).

**TABLE 3:** Results of the logistic regression models of the psychosocial work environment factors and their associations with back pain (*n* = 2202).

Variables	Back pain bivariate models	Back pain multivariable multilevel logistic regression with psychosocial work environment variables (unadjusted model)	Back pain multivariable multilevel logistic regression with psychosocial work environment variables and individual factors (control variables) (adjusted model)
	OR [95% CI]	OR [95% CI]	OR [95% CI]
Intercept		0.46 [0.20, 1.06]	2.75 [0.93, 8.15]
Psychosocial work environment			
Leadership	0.73*** [0.62, 0.86]	0.92 [0.76, 1.10]	1.00 [0.82, 1.21]
Social support from colleagues (scale 0–100)	1.00 [0.99, 1.00]	1.00 [0.99, 1.01]	1.00 [1.00, 1.01]
Role conflicts (scale 0–100)	1.01*** [1.00, 1.01]	1.00 [1.00, 1.01]	1.00 [1.00, 1.01]
Work‐life balance (scale 1–4)	2.19*** [1.80, 2.66]	1.74*** [1.40, 2.17]	1.62*** [1.29, 2.02]
Perceived workload (scale 1–20)	1.14*** [1.10, 1.18]	1.08*** [1.03, 1.12]	1.07*** [1.03. 1.12]
Overtime: yes	1.31** [1.08, 1.59]	0.91 [0.74, 1.12]	0.96 [0.77, 1.18]
Verbal aggression from clients: yes	1.68***[1.38, 2.04]	1.37**[1.12, 1.68]	1.34* [1.09, 1.65]
Offers flexible working schedules	0.76* [0.60, 0.98]	0.79 [0.63, 1.00]	0.82 [0.66, 1.03]
Individual factors			
Age (years)	0.98*** [0.97, 0.99]		0.99*** [0.98, 0.99]
Gender: male^a^	0.56** [0.38, 0.81]		0.49*** [0.33, 0.72]
Gender: non‐binary^a^	0.91 [0.08, 10.48]		0.39 [0.03, 4.95]
Job category: RN^b^	1.00 [0.82, 1.20]		0.86 [0.70, 1.05]
Overall job satisfaction (scale 1–4)	0.53*** [0.44, 0.63]		0.68*** [0.56, 0.84]
Random effect			
Homecare agencies (variance [SD])		0.04 [0.20]	0.03 [0.17]
Effect size			
AIC		2639	2607
Marginal *R* ^2^		0.085	0.102
Conditional *R* ^2^		0.074	0.110

*Note:* α‐level for significance denoted by asterisk(s).

Abbreviations: AIC, Akaike information criterion; CI, confidence interval; OR, odds ratio; RN, registered nurses; SD, standard deviation.

^a^ Female.

^b^ All other nursing and care staff with lower levels of training.

* *p* < 0.05.

** *p* < 0.01.

*** *p* < 0.001.

Higher work‐life balance scores (i.e., poorer work‐life balance), higher perceived workload, and verbal aggression from clients correlated with higher odds of experiencing back pain. The corresponding *R* ^2^ values indicate that the full (adjusted) model performed slightly better at explaining the data than the unadjusted one. However, only approximately 11% of the variance in back pain was explained by the predictors included in the model. The individual factors of age (older) (OR 0.99, 95% CI [0.98, 0.99]), gender (male) (OR 0.49, 95% CI [0.33, 0.72]) and (higher) job satisfaction (OR 0.68, 95% CI [0.56, 0.84]) were negatively associated with back pain (see Table 3).

The sensitivity analyses for back pain as an ordinal outcome did not change the models' overall conclusion; only the job category variable became significant (*p* < 0.01) (see Appendix S4).

We provide a subgroup analysis for the different job categories (‘registered nurses’ and ‘other nursing and care staff’) in Appendix S5.

## 5 Discussion

For this study, we examined the prevalence of burnout and back pain and assessed their associations with psychosocial work environment factors among homecare workers in Switzerland. One in four (26.3%) showed high levels of burnout (≥ 50), while the prevalence of back pain was 68.6%. In line with the WHO healthy workplace model (Burton 2010), poorer work‐life balance, higher perceived workload, and verbal aggression from clients were positively associated with both burnout and back pain. Better leadership and social support from colleagues were negatively associated with burnout. Higher role conflict levels were associated with higher burnout levels. These results have implications for the design of interventions to prevent burnout and back pain among homecare workers.

The mean burnout score (36.0 (SD 18.3)) in our sample (*n* = 2502) was in the same range as in other countries, where the CBI subscale was used (Kristensen et al. 2005): A study conducted among 464 homecare workers in Korea found a mean personal burnout score of 38.6 (SD 18.5) (Jeon et al. 2019). Another study conducted in Denmark reported burnout scores of 43.1 and 32.6 for home help workers respectively in the capital city (*n* = 284) and in a provincial town (*n* = 292) (Kristensen et al. 2005).

Regarding burnout predictors, leadership and social support from colleagues correlated negatively with it. As for leadership, Laschinger et al. (2015) cross‐sectional study suggested that authentic leadership could lead to greater empowerment and occupational coping/self‐efficacy, which could lead, in turn, to lower levels of burnout. Peter et al. (2024) found in a Swiss study comparing healthcare workers in different settings that homecare workers reported the lowest levels of social relations at work and received less feedback from colleagues/supervisors. Such results suggest that interventions to promote social support would be particularly beneficial in homecare, though they might need to be designed differently from those in other settings. Strategies could include regular staff meetings and case discussions, as well as informal after‐work or lunch meetings. As social support from colleagues has been found to bolster both individuals' sense of security and their self‐esteem (Fradelos et al. 2014), it may counteract the negative effects of the work demands.

We found that higher levels of role conflict, poorer work‐life balance, higher perceived workload and verbal aggression from clients correlated positively with burnout. The association between work‐life balance and burnout is consistent with previous findings (Schwartz et al. 2019). This association likely reflects stress resulting from conflicting work and non‐work demands (Sirgy and Lee 2018); that class of conflict may also factor strongly in burnout.

A positive relationship between violence and burnout has also been reported by Sullivan et al. (2022). This agrees with Hobfoll's Conservation of Resources Theory (Hobfoll 1989), which proposes that, when confronted with violence, individuals activate self‐defence mechanisms to protect their personal resources, which can lead to emotional and psychosomatic distress.

Our findings suggest that improving the psychosocial work environment could reduce burnout among workers. This conclusion is consistent with a recent meta‐review's suggestion that organisational‐level interventions (e.g., reducing workload, enhancing teamwork and leadership), whether alone or in combination with individual‐level interventions (e.g., training in stress reduction techniques or communication skills), can reduce burnout (Aust et al. 2023). Strategies for redesigning the psychosocial work environment could include giving workers more influence over their scheduling to improve work‐life balance and increasing workers' influence over the organisation of work, such as through self‐organising teamwork. Introducing participative work group activities to improve communication and team support could improve social support. Training leaders to support workers' well‐being and offering programmes like mindfulness training and stress management can empower workers to handle stressful situations more effectively (Aust et al. 2023).

In our study, more than two‐thirds (68.6%) of homecare workers reported back pain. In comparison, in a study conducted on nursing personnel in a medical centre in China, Yoshimoto et al. (2020) showed that 62.1% (*n* = 668) had experienced low back pain over the previous 4 weeks. In 2022, a national survey of the Swiss population showed 4‐week back pain prevalences of 40.1% in men and 49.9% in women (Federal Statistical Office 2023). As our sample differed from that of the national Swiss study in ways for which we have not controlled, no precise comparison is possible between our results and those of the overall Swiss population.

Still, our findings include evidence that a poorer work‐life balance, a higher perceived workload, and verbal aggression from clients are positively associated with back pain. A poor work‐life balance may result in insufficient time to recover from work, that is, too little time for muscle relaxation and recovery. This supports Min (2022) report of an association between work‐life balance and physical pain in registered nurses working in nursing homes. Regarding the association between back pain and workload, our results support those of a systematic review and meta‐analysis that identified workload, job control and social support as significantly associated with lower back pain (Buruck et al. 2019).

Also, previous studies have linked back pain with verbal aggression from clients (Dhaini et al. 2016). While this relationship's precise mechanism remains unclear, one plausible explanation is that exposure to verbal violence leads to feelings of anxiety and anger, activating a stress response. This, in turn, triggers physiological processes that alter the body's pain processing mechanisms and/or increase muscle tone in ways that precipitate muscle pain (Hauke et al. 2011). Thus, homecare agencies should consider developing policies and strategies to prevent, manage and help workers to cope with verbal aggression from clients.

Interestingly, our model explained only 11% of the variance in back pain, suggesting that other unanalyzed influences, for example, the physical work environment (e.g., the existence of assistive devices, the level of physical load) and other individual factors (e.g., genetic predispositions, muscle strength), play important roles. Nevertheless, our results emphasise the importance of the three identified factors, that is, workload, verbal aggression and work‐life balance. In addition to interventions to improve the psychosocial work environment, innovations such as wearable back support exoskeletons have been developed. In the future, such aids may help to improve healthcare professionals' physical work environment and reduce or even prevent back pain. As another possible strategy, homecare agency‐incentivised exercise programs could also improve muscle strength, thereby preventing or reducing back pain (Bernardelli et al. 2020).

Our findings confirm the need to monitor and implement interventions that improve homecare workers' psychosocial work environment and health. Some countries, including Denmark, have binding regulations requiring employers to improve and regularly monitor psychosocial work environment risk factors, for example, high workload, work‐related violence, and unclear or conflicting work‐related demands (Danish Working Environment Authority 2020).

Thus, interventions to protect workers' health should focus on preventing harm (e.g., assessing and mitigating known risk factors), promoting the positive aspects of work (e.g., by redesigning their work environments) and responding promptly to problems (e.g., via employee assistance programs) (Rugulies et al. 2023).

### 5.1 Strengths and Limitations

One strength of this study is the high response rate from homecare workers and the sample size, which was large enough to produce robust effects. Wherever possible, we used validated measurement instruments, all of which showed good reliability in our sample. Only three items were self‐developed. These three, for which no validated instruments were available for our purposes, were the items on overtime, verbal aggression from clients and the offer of flexible working schedules.

One notable limitation is the study's cross‐sectional design. As this does not indicate the temporal directions of the associations found, no causal inferences are possible. Further, all measures were self‐reported; therefore, biases such as social desirability and recall bias cannot be ruled out. As almost all measures (the one exception being the flexibility of work schedules) were assessed within the same questionnaire, our results could have been affected by common method bias. Moreover, considering that homecare settings differ between countries, and that this study was conducted entirely in Switzerland, its findings may not be generalizable to other healthcare systems. And finally, all data were collected during the COVID‐19 pandemic. This may have influenced workers' perceptions of their psychosocial work environment and health‐related outcomes.

### 5.2 Recommendations for Further Research

Further research—using longitudinal study designs and innovative methods (e.g., mixed methods)—will be necessary to consolidate our understanding of psychosocial work environment factors' impacts on burnout and back pain. Moreover, future research should focus on how to facilitate the successful implementation of guidelines, policies and interventions into the context of homecare. Still more research will be needed to assess the effectiveness of interventions designed to improve the psychosocial work environment. Focusing on the factors identified here as associated, it will be necessary to measure those factors' impacts on homecare workers' health and well‐being.

### 5.3 Implications for Policy and Practice

Our findings on the prevalence of burnout and back pain among homecare workers underscore the need for regular assessment of that group's health status, as well as the urgent need for effective targeted interventions to improve their psychosocial work environment and, consequently, their overall health and well‐being. Alongside other policymakers and key stakeholders, government agencies should raise awareness of the psychosocial work environment's influences on workers' health. They should also provide the necessary structures and conditions to stimulate, support, and guide homecare agencies in implementing strategies to improve their work environments. Ideally, these strategies would include the implementation of initiatives to improve work‐life balance, reduce workload, mitigate verbal aggression from clients and promote effective leadership (e.g., by building homecare leaders' capacities to develop supportive leadership styles and implement healthy workplaces).

Prioritising interventions to promote homecare workers' health and well‐being offers a broad range of advantages. For example, the same conditions that improve workers' psychosocial work environment could also enhance patient safety, facilitate retention of existing employees and make homecare more attractive to new ones, thereby alleviating the increasing workforce shortage.

## 6 Conclusion

The results of this study support arguments that psychosocial work environment factors are important to consider when designing interventions to reduce the prevalence of burnout and back pain among homecare workers. Given the projected increase in demand for homecare services, improving homecare workers' psychosocial work environment should be a key focus of homecare managers, government policymakers and other key stakeholders. In addition to hopefully improving worker retention, initiatives and strategies designed to safeguard and promote this group's health and well‐being will contribute to patient safety, quality of care and the long‐term sustainability of homecare itself.

## Author Contributions

**Tania Martins:** conceptualisation, methodology, formal analysis, writing – original draft, review and editing; **Michael Simon:** conceptualisation, methodology, review and editing; **Nathalie Möckli**, **Carla Meyer‐Massetti**, **Roland Fischer** and **Christine Serdaly:** conceptualisation, review and editing; **Franziska Zúñiga:** conceptualisation, methodology, review and editing. All authors read and approved the final manuscript.

## Disclosure

The authors have checked to make sure that this submission conforms as applicable to the Journal's guidelines. Co‐author M.S. has extensive statistical expertise.

## Conflicts of Interest

The authors declare no conflicts of interest.

## Supporting information


Appendix S1.

Appendix S2.

Appendix S3.

Appendix S4.

Appendix S5.

## Data Availability

Due to the sensitivity of the data analysed in this study, they are not publicly available, but are available from the corresponding author upon reasonable request.

## References

[jan16931-bib-0001] Aust, B. , J. L. Møller , M. Nordentoft , et al. 2023. “How Effective Are Organizational‐Level Interventions in Improving the Psychosocial Work Environment, Health, and Retention of Workers? A Systematic Overview of Systematic Reviews.” Scandinavian Journal of Work, Environment & Health 49, no. 5: 315–329. 10.5271/sjweh.4097. PMC10713994 37158211

[jan16931-bib-0002] Bernardelli, G. , L. Vigna , C. Nava , et al. 2020. “Physical Activity in Healthcare Workers With Low Back Pain: Effects of the Back‐FIT Randomized Trial.” Journal of Occupational and Environmental Medicine 62, no. 6: e245–e249. 10.1097/JOM.0000000000001844. 32097286

[jan16931-bib-0003] Burton, J. 2010. “WHO Healthy Workplace Framework and Model: Background and Supporting Literature and Practice.” https://www.who.int/publications/i/item/who‐healthy‐workplace‐framework‐and‐model.

[jan16931-bib-0004] Buruck, G. , A. Tomaschek , J. Wendsche , E. Ochsmann , and D. Dörfel . 2019. “Psychosocial Areas of Worklife and Chronic Low Back Pain: A Systematic Review and Meta‐Analysis.” BMC Musculoskeletal Disorders 20, no. 1: 480. 10.1186/s12891-019-2826-3. 31653249 PMC6814972

[jan16931-bib-0005] Carpenter, D. , T. Famolaro , S. Hassell , et al. 2017. Patient Safety in the Home: Assessment of Issues, Challenges, and Opportunities. Institute for Healthcare Improvement. https://www.ihi.org/sites/default/files/2023‐10/PatientSafetyInTheHome_2017.pdf.

[jan16931-bib-0006] Cavanaugh, J. E. , and A. A. Neath . 2019. “The Akaike Information Criterion: Background, Derivation, Properties, Application, Interpretation, and Refinements.” WIREs Computational Statistics 11, no. 3: e1460. 10.1002/wics.1460.

[jan16931-bib-0007] Dall'Ora, C. , J. Ball , M. Reinius , and P. Griffiths . 2020. “Burnout in Nursing: A Theoretical Review.” Human Resources for Health 18, no. 1: 41. 10.1186/s12960-020-00469-9. 32503559 PMC7273381

[jan16931-bib-0008] Danish Working Environment Authority . 2020. “Executive Order on Psychosocial Working Environment.” https://at.dk/en/regulations/executive‐orders/psychosocial‐working‐environment‐1406/.

[jan16931-bib-0009] Demerouti, E. , A. B. Bakker , F. Nachreiner , and W. B. Schaufeli . 2001. “The Job Demands‐Resources Model of Burnout.” Journal of Applied Psychology 86, no. 3: 499. 10.1037/0021-9010.86.3.499. 11419809

[jan16931-bib-0010] Dhaini, S. R. , F. Zúñiga , D. Ausserhofer , et al. 2016. “Care Workers Health in Swiss Nursing Homes and Its Association With Psychosocial Work Environment: A Cross‐Sectional Study.” International Journal of Nursing Studies 53: 105–115. 10.1016/j.ijnurstu.2015.08.011. 26363704

[jan16931-bib-0011] Federal Statistical Office . 2022. “Hilfe und Pflege zu Hause.” https://www.bfs.admin.ch/bfs/de/home/statistiken/gesundheit/gesundheitswesen/hilfe‐pflege‐hause.html.

[jan16931-bib-0012] Federal Statistical Office . 2023. “Körperliche Beschwerden, 2022.” https://www.bfs.admin.ch/asset/de/28465284.

[jan16931-bib-0013] Fradelos, E. , S. Mpelegrinos , C. Mparo , et al. 2014. “Burnout Syndrome Impacts on Quality of Life in Nursing Professionals: The Contribution of Perceived Social Support.” Progress in Health Sciences 4, no. 1: 102–109. https://api.semanticscholar.org/CorpusID:54208177.

[jan16931-bib-0014] Hall, L. H. , J. Johnson , I. Watt , A. Tsipa , and D. B. O'Connor . 2016. “Healthcare Staff Wellbeing, Burnout, and Patient Safety: A Systematic Review.” PLoS One 11, no. 7: e0159015. 10.1371/journal.pone.0159015. 27391946 PMC4938539

[jan16931-bib-0015] Hauke, A. , J. Flintrop , E. Brun , and R. Rugulies . 2011. “The Impact of Work‐Related Psychosocial Stressors on the Onset of Musculoskeletal Disorders in Specific Body Regions: A Review and Meta‐Analysis of 54 Longitudinal Studies.” Work & Stress 25, no. 3: 243–256. 10.1080/02678373.2011.614069.

[jan16931-bib-0016] Hegewald, J. , W. Berge , P. Heinrich , et al. 2018. “Do Technical Aids for Patient Handling Prevent Musculoskeletal Complaints in Health Care Workers?—A Systematic Review of Intervention Studies.” International Journal of Environmental Research and Public Health 15, no. 3: 476. 10.3390/ijerph15030476. 29522440 PMC5877021

[jan16931-bib-0017] Hignett, S. , M. Edmunds Otter , and C. Keen . 2016. “Safety Risks Associated With Physical Interactions Between Patients and Caregivers During Treatment and Care Delivery in Home Care Settings: A Systematic Review.” International Journal of Nursing Studies 59: 1–14. 10.1016/j.ijnurstu.2016.02.011. 27222445

[jan16931-bib-0018] Hobfoll, S. E. 1989. “Conservation of Resources. A New Attempt at Conceptualizing Stress.” American Psychologist 44, no. 3: 513. 10.1037//0003-066x.44.3.513. 2648906

[jan16931-bib-0019] Jeon, G. S. , S. J. You , M. G. Kim , Y. M. Kim , and S. I. Cho . 2019. “Psychometric Properties of the Korean Version of the Copenhagen Burnout Inventory in Korean Homecare Workers for Older Adults.” PLoS One 14, no. 8: e0221323. 10.1371/journal.pone.0221323. 31454378 PMC6711598

[jan16931-bib-0020] Kristensen, T. S. , M. Borritz , E. Villadsen , and K. B. Christensen . 2005. “The Copenhagen Burnout Inventory: A New Tool for the Assessment of Burnout.” Work & Stress 19, no. 3: 192–207. 10.1080/02678370500297720.

[jan16931-bib-0021] Laschinger, H. K. , L. Borgogni , C. Consiglio , and E. Read . 2015. “The Effects of Authentic Leadership, Six Areas of Worklife, and Occupational Coping Self‐Efficacy on New Graduate Nurses' Burnout and Mental Health: A Cross‐Sectional Study.” International Journal of Nursing Studies 52, no. 6: 1080–1089. 10.1016/j.ijnurstu.2015.03.002. 25801311

[jan16931-bib-0022] Min, D. 2022. “Effects of Resilience, Burnout, and Work‐Related Physical Pain on Work‐Life Balance of Registered Nurses in South Korean Nursing Homes: A Cross‐Sectional Study.” Medicine 101, no. 30: e29889. 10.1097/md.0000000000029889. 35905217 PMC9333544

[jan16931-bib-0023] Möckli, N. , M. Simon , C. Meyer‐Massetti , et al. 2021. “Factors Associated With Homecare Coordination and Quality of Care: A Research Protocol for a National Multi‐Center Cross‐Sectional Study.” BMC Health Services Research 21, no. 1: 306. 10.1186/s12913-021-06294-7. 33823850 PMC8025374

[jan16931-bib-0024] Nakagawa, S. , and H. Schielzeth . 2013. “A General and Simple Method for Obtaining *R* ^2^ From Generalized Linear Mixed‐Effects Models.” Methods in Ecology and Evolution 4, no. 2: 133–142. 10.1111/j.2041-210x.2012.00261.x.

[jan16931-bib-0025] Patwardhan, V. , G. F. Gil , A. Arrieta , et al. 2024. “Differences Across the Lifespan Between Females and Males in the Top 20 Causes of Disease Burden Globally: A Systematic Analysis of the Global Burden of Disease Study 2021.” Lancet Public Health 9, no. 5: e282–e294. 10.1016/S2468-2667(24)00053-7. 38702093 PMC11080072

[jan16931-bib-0026] Peter, K. A. , C. Voirol , S. Kunz , et al. 2024. “Factors Associated With Health Professionals' Stress Reactions, Job Satisfaction, Intention to Leave and Health‐Related Outcomes in Acute Care, Rehabilitation and Psychiatric Hospitals, Nursing Homes and Home Care Organisations.” BMC Health Services Research 24, no. 1: 269. 10.1186/s12913-024-10718-5. 38431643 PMC10909269

[jan16931-bib-0027] Robstad, A. G. , and R. H. Westgaard . 2014. “Perceived Occupational Exposures of Home Care Workers and the Association to General Tension, Shoulder‐Neck and Low Back Pain.” Work 49, no. 4: 723–733. 10.3233/wor-131710. 24004770

[jan16931-bib-0028] Rugulies, R. , B. Aust , B. A. Greiner , et al. 2023. “Work‐Related Causes of Mental Health Conditions and Interventions for Their Improvement in Workplaces.” Lancet 402, no. 10410: 1368–1381. 10.1016/S0140-6736(23)00869-3. 37838442

[jan16931-bib-0029] Santos, P. , R. Martins , and F. Serranheira . 2016. “Prevalência da dor Lombar em Enfermeiros em Contexto Hospitalar.” Gestão e Desenvolvimento 24: 161–171. https://hdl.handle.net/10400.19/4442.

[jan16931-bib-0030] Schwartz, S. P. , K. C. Adair , J. Bae , et al. 2019. “Work‐Life Balance Behaviours Cluster in Work Settings and Relate to Burnout and Safety Culture: A Cross‐Sectional Survey Analysis.” BMJ Quality and Safety 28, no. 2: 142–150. 10.1136/bmjqs-2018-007933. PMC6365921 30309912

[jan16931-bib-0031] Sirgy, M. J. , and D.‐J. Lee . 2018. “Work‐Life Balance: An Integrative Review.” Applied Research in Quality of Life 13, no. 1: 229–254. 10.1007/s11482-017-9509-8.

[jan16931-bib-0032] Sullivan, D. , K. M. White , and C. Frazer . 2022. “Factors Associated With Burnout in the United States Versus International Nurses.” Nursing Clinics of North America 57, no. 1: 29–51. 10.1016/j.cnur.2021.11.003. 35236607

[jan16931-bib-0033] Thompson, C. G. , R. S. Kim , A. M. Aloe , and B. J. Becker . 2017. “Extracting the Variance Inflation Factor and Other Multicollinearity Diagnostics From Typical Regression Results.” Basic and Applied Social Psychology 39, no. 2: 81–90. 10.1080/01973533.2016.1277529.

[jan16931-bib-0034] U.S. Bureau of Labor Statistics . 2021. “Injuries, Illness, and Fatalities: Table 1. Incidence Rates of Nonfatal Occupational Injuries and Illnesses by Industry and Case Types, 2020.” https://www.bls.gov/web/osh/summ1_00.htm.

[jan16931-bib-0035] Woo, T. , R. Ho , A. Tang , and W. Tam . 2020. “Global Prevalence of Burnout Symptoms Among Nurses: A Systematic Review and Meta‐Analysis.” Journal of Psychiatric Research 123: 9–20. 10.1016/j.jpsychires.2019.12.015. 32007680

[jan16931-bib-0036] World Health Organization . 2021. “International Classification of Diseases 11th Revision (ICD‐11).” https://icd.who.int/en.

[jan16931-bib-0037] World Health Organization . 2022. “Occupational Health: Health Workers.” https://www.who.int/news‐room/fact‐sheets/detail/occupational‐health‐‐health‐workers.

[jan16931-bib-0038] Yoshimoto, T. , H. Oka , H. Ochiai , et al. 2020. “Presenteeism and Associated Factors Among Nursing Personnel With Low Back Pain: A Cross‐Sectional Study.” Journal of Pain Research 13: 2979–2986. 10.2147/jpr.S269529. 33239906 PMC7682615

