# Supplementary material for: Burnout and Back Pain and Their Associations With Homecare Workers' Psychosocial Work Environment—A National Multicenter Cross‐Sectional Study
Source: J Adv Nurs. 2025 Apr 2;82(2):1253–64. doi: 10.1111/jan.16931 (PMC12810604; doi:10.1111/jan.16931)
Supplement: Supplementary file 1 — Appendix S1. [file JAN-82-1253-s001.docx]

**Appendix A**

**References**

**Table A1**

*References of the instruments and R packages used*

| **Instrument or R package** | **Reference** |
| --- | --- |
| Copenhagen Burnout Inventory (CBI) | Kristensen, T. S., Borritz, M., Villadsen, E., & Christensen, K. B. (2005). The Copenhagen Burnout Inventory: A new tool for the assessment of burnout. *Work & Stress*, *19*(3), 192–207. <http://doi.org/10.1080/02678370500297720> |
| Federal Statistical Office’s Swiss Health Survey | Federal Statistical Office. (2019). *Schweizerische Gesundheitsbefragung*. <https://www.bfs.admin.ch/bfs/de/home/statistiken/gesundheit/erhebungen/sgb.assetdetail.7606607.html> |
| Practice Environment Scale of the Nursing Work Index’s (PES-NWI’s) Nurse Manager Ability, Leadership and Support of Nurses subscale | Lake, E. T. (2002). Development of the practice environment scale of the Nursing Work Index. *Res Nurs Health*, *25*(3), 176–188. <https://doi.org/10.1002/nur.10032> |
| Copenhagen Psychosocial Questionnaire (COPSOQ) | Pejtersen, J. H., Kristensen, T. S., Borg, V., & Bjorner, J. B. (2010). The second version of the Copenhagen Psychosocial Questionnaire. *Scand J Public Health*, *38*(3 Suppl), 8–24. <https://doi.org/10.1177/1403494809349858> |
| National Aeronautics and Space Administration Task Load Index (NASA-TLX) | Hart, S. G., & Staveland, L. E. (1988). Development of NASA-TLX (Task Load Index): Results of Empirical and Theoretical Research. *Advances in Psychology*, *52*, 139–183. <https://doi.org/10.1016/S0166-4115(08)62386-9> |
| Work-life climate scale | Sexton, J. B., Helmreich, R. L., Neilands, T. B., Rowan, K., Vella, K., Boyden, J., . . . Thomas, E. J. (2006). The safety attitudes questionnaire: psychometric properties, benchmarking data, and emerging research. *BMC Health Services Research*, *6*(1), 44. <https://doi.org/10.1186/1472-6963-6-44> |
| Registered Nurse Forecasting (RN4CAST) study | Sermeus, W., Aiken, L. H., Van den Heede, K., Rafferty, A. M., Griffiths, P., Moreno-Casbas, M. T., . . . Zikos, D. (2011). Nurse forecasting in Europe (RN4CAST): Rationale, design and methodology. *BMC Nurs*, *10*, 6. <https://doi.org/10.1186/1472-6955-10-6> |
| “rptR” package | Stoffel, M. A., Nakagawa, S., & Schielzet, H. (2017). rptR: Repeatability estimation and variance decomposition by generalized linear mixed-effects models. *Methods in Ecology and Evolution*, *8*(11). <https://doi.org/10.1111/2041-210X.12797> |
| “lme4” package | Bates, D., Mächler, M., Bolker, B., & Walker, S. (2015). Fitting Linear Mixed-Effects Models Using lme4. *Journal of Statistical Software*, *67*(1), 1–48. <https://doi.org/10.18637/jss.v067.i01> |
| “performance” package | Lüdecke, D., Ben-Shachar, M. S., Patil, I., Waggoner, P., & Makowski, D. (2021). {performance}: An {R} Package for Assessment, Comparison and Testing of Statistical Models. *Journal of Open Source Software*, *6*(60), 3139. <https://doi.org/10.21105/joss.03139> |
| R version 4.2.3 | R Core Team. (2023). *R: A Language and Environment for Statistical Computing*. In <https://R-project.org/> |
| “ordinal” package | Christensen, R. H. B. (2023). *ordinal---Regression Models for Ordinal Data*. <https://CRAN.R-project.org/package=ordinal> |
