# Supplementary material for: Burnout and Back Pain and Their Associations With Homecare Workers' Psychosocial Work Environment—A National Multicenter Cross‐Sectional Study
Source: J Adv Nurs. 2025 Apr 2;82(2):1253–64. doi: 10.1111/jan.16931 (PMC12810604; doi:10.1111/jan.16931)
Supplement: Supplementary file 2 — Appendix S2. [file JAN-82-1253-s002.docx]

**Appendix B**

**Flow chart of the in- and excluded respondents**

**Initial dataset** (n=3223))

**Inclusion criteria not met** (n=638)

Job category (administration, apprentices, trainees) (n=253)

Leadership position (n=373)

Work domain: Employees not involved in the provision of care or housekeeping (n=12)

**Dataset for analysis** (n=2514))

**Inclusion criteria not available** (n=71)

Job category (administration, apprentices, trainees) (n=24)

Leadership position (n=41)

Work domain (n=6)

**Figure B1**

*Flow chart showing the number of homecare workers included in the analyses*
