# Supplementary material for: Burnout and Back Pain and Their Associations With Homecare Workers' Psychosocial Work Environment—A National Multicenter Cross‐Sectional Study
Source: J Adv Nurs. 2025 Apr 2;82(2):1253–64. doi: 10.1111/jan.16931 (PMC12810604; doi:10.1111/jan.16931)
Supplement: Supplementary file 3 — Appendix S3. [file JAN-82-1253-s004.docx]

**Appendix C**

**Burnout – Subgroup analysis**

**Table C1**

*Results of the linear regression models of the psychosocial work environment factors and their associations with burnout for job category ‘registered nurses’ (n=889)*

|  |  | Burnout bivariate models |  | Burnout multivariable model with psychosocial work environment variables (unadjusted model) |  | Burnout multivariable model with psychosocial work environment variables and individual factors (control variables) (adjusted model) |
| --- | --- | --- | --- | --- | --- | --- |
| **Variables** |  | *B* [95% CI] |  | *B* [95% CI] |  | *B* [95% CI] |
| **Intercept** |  |  |  | 6.77 [-2.13, 15.67] |  | 23.13*** [12.02, 34.25] |
| **Psychosocial work environment** |  |  |  |  |  |  |
| Leadership |  | -9.32*** [-11.22, -7.42] |  | -3.55*** [-5.36, -1.73] |  | -2.74** [-4.61, -0.88] |
| Social support from colleagues  (scale 0-100) |  | -0.14*** [-0.20, -0.07] |  | -0.04 [-0.09, -0.01] |  | -0.04 [-0.09, -0.01] |
| Role conflicts (scale 0-100) |  | 0.26*** [0.21, 0.31] |  | 0.09*** [ 0.04, 0.14] |  | 0.07** [0.02, 0.12] |
| Work-life balance (scale 1-4) |  | 17.21*** [15.20, 19.23] |  | 10.96***[8.81, 13.11] |  | 10.42*** [8.27, 12.56] |
| Perceived workload (scale 1-20) |  | 3.32*** [2.90, 3.74] |  | 1.76*** [1.32, 2.20] |  | 1.62*** [1.17, 2.06] |
| Overtime: yes |  | 9.08*** [6.35, 11.81] |  | 0.90 [-1.50, 3.30] |  | 0.99 [-1.39, 3.36] |
| Verbal aggression from clients: yes |  | 8.55*** [5.79, 11.31] |  | 3.70** [1.38, 6.02] |  | 3.53** [1.23, 5.82] |
| Offers flexible working schedules |  | -2.61 [-6.11, 0.90] |  | -0.84 [-3.29, 1.61] |  | -0.60 [-3.05, 1.85] |
| **Individual factors** |  |  |  |  |  |  |
| Age (years) |  | -0.15** [-0.26, -0.05] |  |  |  | -0.11* [-0.19, -0.02] |
| Gender: male^a^ |  | -1.98 [-6.27, 2.31] |  |  |  | -3.32 [-6.76, 0.11] |
| Overall job satisfaction (scale 1-4) |  | -11-55*** [-13.51, -9.59] |  |  |  | -3.68*** [-5.63, -1.73] |
| **Random effect** |  |  |  |  |  |  |
| Homecare agencies (variance [SD]) |  |  |  | 5.07 [2.25] |  | 5.41 [2.33] |
| **Effect size** |  |  |  |  |  |  |
| AIC |  |  |  | 7299 |  | 7274 |
| Marginal R^2^ |  |  |  | 0.368 |  | 0.385 |
| Conditional R^2^ |  |  |  | 0.383 |  | 0.401 |
| *Note*. α-level for significance: **p* < .05. ***p* < .01. ****p* < .001  Abbreviations*:* CI, Confidence interval; *B*, unstandardized regression coefficients; RN, Registered nurses; SD, Standard deviation; AIC, Akaike information criterion  Reference categories: ^a^ female. | | | | | | |

**Table C2**

*Results of the linear regression models of the psychosocial work environment factors and their associations with burnout for the job category ‘other nursing and care staff’ (n=1335)*

|  |  | Burnout bivariate models |  | Burnout multivariable model with psychosocial work environment variables (unadjusted model) |  | Burnout multivariable model with psychosocial work environment variables and individual factors (control variables) (adjusted model) |
| --- | --- | --- | --- | --- | --- | --- |
| **Variables** |  | *B* [95% CI] |  | *B* [95% CI] |  | *B* [95% CI] |
| **Intercept** |  |  |  | 18.46*** [11.34, 25.57] |  | 46.88*** [12.02, 34.25] |
| **Psychosocial work environment** |  |  |  |  |  |  |
| Leadership |  | -8.93*** [-10.54, -7.32] |  | -4.39*** [-5.93, -2.84] |  | -2.47** [-4.61, -0.88] |
| Social support from colleagues  (scale 0-100) |  | -0.11*** [-0.15, -0.06] |  | -0.04* [-0.08, -0.00] |  | -0.03 [-0.09, 0.01] |
| Role conflicts (scale 0-100) |  | 0.18*** [0.14, 0.22] |  | 0.03 [ 0.01, 0.07] |  | 0.03 [0.02, 0.12] |
| Work-life balance (scale 1-4) |  | 16.59*** [14.95, 18.23] |  | 12.16*** [10.42, 13.91] |  | 10.44*** [8.27, 12.56] |
| Perceived workload (scale 1-20) |  | 2.45*** [2.14, 2.75] |  | 1.36*** [1.04, 1.66] |  | 1.24*** [1.17, 2.06] |
| Overtime: yes |  | 6.42*** [4.49, 8.35] |  | -0.17 [-1.90, 1.56] |  | -0.06 [-1.39, 3.36] |
| Verbal aggression from clients: yes |  | 6.31*** [4.32, 8.31] |  | 1.40 [-0.34, 3.14] |  | 0.89 [1.23, 5.82] |
| Offers flexible working schedules |  | -4.12* [-7.40, -0.84] |  | -3.21* [-5.76, -0.68] |  | -2.51* [-3.05, 1.85] |
| **Individual factors** |  |  |  |  |  |  |
| Age (years) |  | -0.24*** [-0.32, -0.16] |  |  |  | -0.17*** [-0.19, -0.02] |
| Gender: male^a^ |  | -5.19* [-10.31, -0.07] |  |  |  | -6.48** [-6.76, 0.11] |
| Overall job satisfaction (scale 1-4) |  | -14.04*** [-15.75, -12.32] |  |  |  | -7.52*** [-5.63, -1.73] |
| **Random effect** |  |  |  |  |  |  |
| Homecare agencies (variance [SD]) |  |  |  | 11.69 [3.42] |  | 9.62 [3.10] |
| **Effect size** |  |  |  |  |  |  |
| AIC |  |  |  | 11003 |  | 10839 |
| Marginal R^2^ |  |  |  | 0.323 |  | 0.378 |
| Conditional R^2^ |  |  |  | 0.358 |  | 0.407 |
| *Note*. α-level for significance: **p* < .05. ***p* < .01. ****p* < .001  Abbreviations*:* CI, Confidence interval; *B*, unstandardized regression coefficients; RN, Registered nurses; SD, Standard deviation; AIC, Akaike information criterion  Reference categories: ^a^ female. | | | | | | |
