# Supplementary material for: Burnout and Back Pain and Their Associations With Homecare Workers' Psychosocial Work Environment—A National Multicenter Cross‐Sectional Study
Source: J Adv Nurs. 2025 Apr 2;82(2):1253–64. doi: 10.1111/jan.16931 (PMC12810604; doi:10.1111/jan.16931)
Supplement: Supplementary file 4 — Appendix S4. [file JAN-82-1253-s003.docx]

**Appendix D**

**Sensitivity analysis**

As we dichotomized the back pain outcome for the analysis, we ran an ordinal logistic regression model as a sensitivity analysis.

The results of the ordinal logistic regression model for back pain are shown in Table D1.

**Table D1**

*Back pain ordinal logistic regression model (n=2202)*

|  |  | Back pain multivariable ordinal logistic regression model with psychosocial work environment variables (unadjusted model) |  | Back pain multivariable ordinal logistic regression model with psychosocial work environment variables and individual factors (control variables) (adjusted model) |
| --- | --- | --- | --- | --- |
| **Variables** |  | $\mathrm{OR}$ [95% CI] |  | $\mathrm{OR}$ [95% CI] |
| **Intercept** |  |  |  |  |
| **1** (‘not at all’**\| 2** (‘a little’) |  | 2.64* [1.26, 5.54] |  | 0.42 [0.16, 1.09] |
| **2** (‘a little’) **\| 3 (**‘strongly’) |  | 30.69*** [14.42, 65.31] |  | 5.14*** [1.98, 13.32] |
| **Psychosocial work environment** |  |  |  |  |
| Leadership |  | 0.88 [0.76, 1.03] |  | 0.95 [0.80, 1.15] |
| Social support from colleagues (scale 0-100) |  | 1.00 [1.00, 1.01] |  | 1.00 [1.00, 1.01] |
| Role conflicts (scale 0-100) |  | 1.00 [1.00, 1.00] |  | 1.00 [1.00, 1.01] |
| Work-life balance (scale 1-4) |  | 1.76*** [1.47, 2.12] |  | 1.64*** [1.36, 1.97] |
| Perceived workload (scale 1-20) |  | 1.11*** [1.07, 1.15] |  | 1.11*** [1.07. 1.15] |
| Overtime: yes |  | 0.92 [0.77, 1.11] |  | 0.98 [0.82, 1.19] |
| Verbal aggression from clients: yes |  | 1.26* [1.04, 1.51] |  | 1.23* [1.02, 1.49] |
| Offers flexible working schedules |  | 0.83 [0.65, 1.05] |  | 0.86 [0.68, 1.08] |
| **Individual factors** |  |  |  |  |
| Age (years) |  |  |  | 0.98*** [0.98, 0.99] |
| Gender: male^a^ |  |  |  | 0.50*** [0.34, 0.71] |
| Gender: non-binary^a^ |  |  |  | 0.31 [0.03, 3.02] |
| Job category: RN^b^ |  |  |  | 0.76** [0.64, 0.91] |
| Overall job satisfaction  (scale 1-4) |  |  |  | 0.71*** [0.59, 0.84] |
| **Random effect** |  |  |  |  |
| Homecare agencies (variance [SD]) |  | 0.08 [0.29] |  | 0.08 [0.28] |
| **Effect size** |  |  |  |  |
| AIC |  | 4333 |  | 4282 |
| Marginal R^2^ |  | 0.087 |  | 0.116 |
| Conditional R^2^ |  | 0.110 |  | 0.137 |

*Notes*. α-level for significance: **p* < .05. ***p* < .01. ****p* < .001

Abbreviations: OR, Odds ratio; CI, Confidence interval; RN, Registered nurses; SD, Standard deviation; AIC, Akaike information criterion

Reference categories: ^a^ female; ^b^ all other nursing and care staff with lower levels of training
