# Supplementary material for: Burnout and Back Pain and Their Associations With Homecare Workers' Psychosocial Work Environment—A National Multicenter Cross‐Sectional Study
Source: J Adv Nurs. 2025 Apr 2;82(2):1253–64. doi: 10.1111/jan.16931 (PMC12810604; doi:10.1111/jan.16931)
Supplement: Supplementary file 5 — Appendix S5. [file JAN-82-1253-s005.docx]

**Appendix E**

**Back pain – Subgroup analysis**

**Table E1**

*Results of the logistic regression models of the psychosocial work environment factors and their associations with back pain for the job category ‘registered nurses’ (n=888)*

|  |  | Back pain bivariate models |  | Back pain multivariable multilevel logistic regression with psychosocial work environment variables (unadjusted model) |  | Back pain multivariable multilevel logistic regression with psychosocial work environment variables and individual factors (control variables) (adjusted model) |
| --- | --- | --- | --- | --- | --- | --- |
| **Variables** |  | $\mathrm{OR}$ [95% CI] |  | $\mathrm{OR}$ [95% CI] |  | $\mathrm{OR}$ [95% CI] |
| **Intercept** |  |  |  | 0.89 [0.22, 3.59] |  | 4.17 [0.71, 24.54] |
| **Psychosocial work environment** |  |  |  |  |  |  |
| Leadership |  | 0.64*** [0.49, 0.82] |  | 0.82 [0.62, 1.11] |  | 0.87 [0.65, 1.18] |
| Social support from colleagues  (scale 0-100) |  | 0.99 [0.98, 1.00] |  | 1.00 [0.99, 1.00] |  | 1.00 [0.99, 1.00] |
| Role conflicts (scale 0-100) |  | 1.01** [1.00, 1.02] |  | 1.00 [0.99, 1.01] |  | 1.00 [0.99, 1.01] |
| Work-life balance (scale 1-4) |  | 1.69*** [1.26, 2.28] |  | 1.21 [0.86, 1.71] |  | 1.14 [0.81, 1.61] |
| Perceived workload (scale 1-20) |  | 1.19*** [1.12, 1.26] |  | 1.16*** [1.08, 1.24] |  | 1.15*** [1.07. 1.24] |
| Overtime: yes |  | 1.03 [0.73, 1.44] |  | 0.69 [0.48, 1.00] |  | 0.71 [0.49, 1.03] |
| Verbal aggression from clients:  yes |  | 1.67** [1.20, 2.32] |  | 1.37 [0.97, 1.93] |  | 1.33 [0.94, 1.89] |
| Offers flexible working schedules |  | 0.77 [0.54, 1.09] |  | 0.83 [0.60, 1.17] |  | 0.84 [0.60, 1.18] |
| **Individual factors** |  |  |  |  |  |  |
| Age (years) |  | 0.99* [0.97, 1.00] |  |  |  | 0.99 [0.97, 1.00] |
| Gender: male^a^ |  | 0.55* [0.34, 0.90] |  |  |  | 0.49** [0.29, 0.81] |
| Overall job satisfaction (scale 1-4) |  | 0.53*** [0.41, 0.70] |  |  |  | 0.73 [0.54, 1.00] |
| **Random effect** |  |  |  |  |  |  |
| Homecare agencies (variance [SD]) |  |  |  | 0.03 [0.17] |  | 0.03 [0.17] |
| **Effect size** |  |  |  |  |  |  |
| AIC |  |  |  | 1064 |  | 1057 |
| Marginal R^2^ |  |  |  | 0.083 |  | 0.153 |
| Conditional R^2^ |  |  |  | 0.091 |  | 0.161 |
| *Note*. α-level for significance: **p* < .05. ***p* < .01. ****p* < .001  Abbreviations: OR, Odds ratio; CI, Confidence interval; SD, Standard deviation; AIC, Akaike information criterion  Reference categories: ^a^ female. | | | | | | |

**Table E2**

*Results of the logistic regression models of the psychosocial work environment factors and their associations with back pain for the job category ‘other nursing and care staff’ (n=1314)*

|  |  | Back pain bivariate models |  | Back pain multivariable multilevel logistic regression with psychosocial work environment variables (unadjusted model) |  | Back pain multivariable multilevel logistic regression with psychosocial work environment variables and individual factors (control variables) (adjusted model) |
| --- | --- | --- | --- | --- | --- | --- |
| **Variables** |  | $\mathrm{OR}$ [95% CI] |  | $\mathrm{OR}$ [95% CI] |  | $\mathrm{OR}$ [95% CI] |
| **Intercept** |  |  |  | 0.26* [0.09, 0.77] |  | 1.80 [0.44, 7.38] |
| **Psychosocial work environment** |  |  |  |  |  |  |
| Leadership |  | 0.79* [0.64, 0.98] |  | 1.01 [0.79, 1.28] |  | 1.12 [0.87, 1.44] |
| Social support from colleagues  (scale 0-100) |  | 1.00 [0.99, 1.00] |  | 1.00 [1.00, 1.01] |  | 1.00 [1.00, 1.01] |
| Role conflicts (scale 0-100) |  | 1.01** [1.00, 1.01] |  | 1.00 [1.00, 1.01] |  | 1.00 [1.00, 1.01] |
| Work-life balance (scale 1-4) |  | 2.73*** [2.09, 3.57] |  | 2.25*** [1.68, 3.02] |  | 2.04*** [1.51, 2.75] |
| Perceived workload (scale 1-20) |  | 1.11*** [1.07, 1.16] |  | 1.04 [1.00, 1.09] |  | 1.04 [1.00, 1.09] |
| Overtime: yes |  | 1.56*** [1.22, 1.99] |  | 1.11 [0.85, 1.45] |  | 1.13 [0.87, 1.48] |
| Verbal aggression from clients:  yes |  | 1.71*** [1.34, 2.19] |  | 1.37* [1.06, 1.78] |  | 1.34* [1.03, 1.73] |
| Offers flexible working schedules |  | 0.76 [0.55, 1.04] |  | 0.75 [0.55, 1.02] |  | 0.78 [0.59, 1.04] |
| **Individual factors** |  |  |  |  |  |  |
| Age (years) |  | 0.98*** [0.97, 0.99] |  |  |  | 0.98** [0.97, 0.99] |
| Gender: male^a^ |  | 0.57 [0.31, 1.04] |  |  |  | 0.46* [0.24, 0.86] |
| Overall job satisfaction (scale 1-4) |  | 0.52*** [0.41, 0.66] |  |  |  | 0.63*** [0.48, 0.83] |
| **Random effect** |  |  |  |  |  |  |
| Homecare agencies (variance [SD]) |  |  |  | 0.07 [0.26] |  | 0.03 [0.17] |
| **Effect size** |  |  |  |  |  |  |
| AIC |  |  |  | 1570 |  | 1552 |
| Marginal R^2^ |  |  |  | 0.094 |  | 0.125 |
| Conditional R^2^ |  |  |  | 0.113 |  | 0.133 |
| *Note*. α-level for significance: **p* < .05. ***p* < .01. ****p* < .001  Abbreviations: OR, Odds ratio; CI, Confidence interval; SD, Standard deviation; AIC, Akaike information criterion  Reference categories: ^a^ female. | | | | | | |
